# The Effects of Health Anxiety and Litigation Potential on Symptom Endorsement, Cognitive Performance, and Physiological Functioning in the Context of a Food and Drug Administration Drug Recall Announcement

**DOI:** 10.3389/fpsyg.2022.818724

**Published:** 2022-06-13

**Authors:** Len Lecci, Gary Ryan Page, Julian R. Keith, Sarah Neal, Ashley Ritter

**Affiliations:** Department of Psychology, University of North Carolina Wilmington, Wilmington, NC, United States

**Keywords:** health anxiety, drug recall, malingering, side-effects, litigation

## Abstract

Drug recalls and lawsuits against pharmaceutical manufacturers are accompanied by announcements emphasizing harmful drug side-effects. Those with elevated health anxiety may be more reactive to such announcements. We evaluated whether health anxiety and financial incentives affect subjective symptom endorsement, and objective outcomes of cognitive and physiological functioning during a mock drug recall. Hundred and sixty-one participants reported use of over-the-counter pain medications and presented with a fictitious medication recall via a mock Food and Drug Administration (FDA) website. The opportunity to join a class-action lawsuit was manipulated. We assessed health anxiety, recalled drug usage, blood pressure, heart rate, and performance on a computerized Trail Making Test (TMT). Symptom endorsement was strongly predicted by health anxiety. When combined, three health anxiety measures explained 28.5% variance (Cohen’s *d* = 1.26). These effects remain strong after controlling for depression and anxiety. Litigation condition did not predict symptom endorsement. Blood pressure and heart rate were modestly predicted by health anxiety, but not by litigation condition. TMT performance was consistently predicted by health anxiety, with higher scores associated with poorer performance. Although there were no main effects for litigation, interactions consistently emerged for the TMT, with generally poorer performance for those with higher health anxiety in the non-litigation condition; whereas health anxiety was unrelated to performance for the litigation condition. All but one participant joined the litigation when given the opportunity, despite a healthy sample and minimal use of pain medication. Subsequent data from 67 individuals with no mention of the FDA scenario or litigation showed that health anxiety still significantly predicts symptom endorsement (12.6% variance), but the explained variance is less than half that obtained in the FDA scenario. The findings suggest that health anxiety plays a significant role in adverse symptom reporting, beyond anxiety or depression, and this effect is independent of the presence of the FDA recall. The lack of differences for health anxiety and symptom endorsement between litigation and non-litigation conditions rules out malingering. Although it is general practice in drug recalls to list potential adverse side effects caused by medications, this may elicit unintended symptom experiences and health anxious individuals may be more susceptible.

## Introduction

Individuals with pre-existing high levels of health anxiety may be particularly susceptible to reporting symptoms and side effects when exposed to information about adverse drug effects. Heightened sensitivity to health-related stimuli is part and parcel of the DSM-5 (American Psychiatric Association, 2013) criteria for diagnosing a health anxiety disorder (e.g., Lees et al., 2005). However, we know little about whether health anxiety, in general, is a factor in responsiveness to health threatening information about drugs; as would occur in drug recall announcements and publicity about lawsuits against pharmaceutical companies. It is also unclear the extent to which potential litigation could affect responses to a drug recall.

For the current study, we created a simulated FDA recall of widely used over-the-counter medications, controlling information related to adverse effects and experimentally manipulating the potential for financial compensation (litigation). We also measured health anxiety and examined its predictive potential, along with that of litigation, and their interaction, with respect to three outcome variables; (1) self-reported symptom endorsement, (2) cognitive performance, and (3) physiological functioning, in order to gauge the consequences for both subjective and objective outcomes. Finally, we also collected a control condition to assess self-reported symptoms and health anxiety outside of the Food and Drug Administration (FDA) context. This experimentally controlled context permitted an analysis of variables that often cannot be differentiated in a naturalistic situation.

### Health Anxiety

Individuals with low levels of health anxiety are generally less likely to consider themselves at risk for adverse health events. Indeed, most people have an optimistic bias regarding health risks (Weinstein, 1984, 1987). Individuals with high health anxiety, however, tend to *believe* they are unhealthy, and endorse more symptoms of illness (e.g., Pennebaker, 1982; Watson and Pennebaker, 1989; Ellington and Wiebe, 1999; Feldman et al., 1999). Health anxious individuals may adopt illness beliefs more quickly and seek out information to validate their negative health beliefs (e.g., Barsky and Klerman, 1983; Kellner, 1986; Warwick, 1989; Cioffi, 1991; for a broader etiological account, see Cisler and Koster, 2010). Research also suggests that measures of health anxiety capture a health content-specific version of the broader construct of negative affect, and the former relates more strongly to the endorsement of physical symptoms (Lecci et al., 1996). It is also the case that when individuals are in a situation/context that itself can elevate health anxious responding, symptom reporting may be especially exacerbated for those already predisposed to experiencing health anxiety (Lecci and Cohen, 2002). Because information contained in drug recall publicity emphasizes adverse, health-threatening effects, we hypothesize that health anxiety will predict symptom endorsement in the simulated drug recall. Moreover, in keeping with previous research (e.g., Lecci and Cohen, 2002) we hypothesize that the association with health anxiety will either be non-existent or not as pronounced in the absence of a potentially health threatening context (i.e., when there is no FDA drug recall).

### The Influence of Monetary Incentives on Behavior

Monetary incentives are powerful motivators of behavior (e.g., Benabou and Tirole, 2006). It is therefore not surprising that increased symptom endorsement is seen among those seeking financial compensation through litigation (Rohling et al., 1995). This can be reflected in the concept of “compensation neurosis,” which is defined as the exaggeration of symptoms resulting from the opportunity to obtain financial reward through legal compensation (Hall and Hall, 2012). The field of neuroeconomics also provides evidence that financial incentives influence brain activity in brain systems associated with expectancy (placebo) effects (Scott et al., 2007), suggesting that financial incentives may influence actual symptom experience.

Individuals also can be motivated to malinger (feigning symptoms for external gain) without experiencing deleterious consequences of exposure to the drug. In neuropsychological settings, estimated rates of malingering range from 15 to 64% according to a meta-analysis that included eleven studies that provided data on malingering (Heaton et al., 1978; Trueblood and Schmidt, 1993; Larrabee, 2003). The detection of malingering often utilizes objective measures, such as assessments of cognitive performance, in addition to the information derived from self-report measures, but it is the objective performance-based measures that can provide the more conclusive findings (e.g., performing significantly below chance on performance validity measures is considered a very strong indicator of malingering, as the individual would have to know the correct response and choose the incorrect alternative in order to score significantly below chance; e.g., Rogers and Bender, 2013). Malingering is also associated with an “amplified presentation” of symptoms (i.e., more symptoms relative to genuine experiences of pathology and perhaps more than those with higher health anxiety), including endorsing large numbers of symptoms, high symptom severity, and endorsement of erroneous symptom stereotypes (Walczyk et al., 2018). The literature is clear in illustrating that malingering is associated with elevating symptom reporting and intentional underperformance on objective cognitive measures for those involved in litigation It is also likely that people experiencing more symptoms (physical and psychological) and functional consequences (marked by underperformance on cognitive measures) are more likely to litigate for compensation (see Samra and Koch, 2002). Thus, differentiating malingering from legitimate symptom experience, or from a health anxious response in people who have taken a drug and report adverse reactions is notoriously difficult, and this has proven to be the case even for trained medical professionals (Bellamy, 1997). Given the widespread publicity associated with drug recalls and the involvement of a psychologically diverse population, similar large-scale challenges are likely to exist in this context. We predict that when individuals are presented with the opportunity to participate in litigation during a simulated drug recall, they will do so regardless of their health anxiety. Moreover, in keeping with research examining the influence of external contingencies on malingering in college students (e.g., Boskovic, 2020), we predict that the litigation condition will result in greater symptom endorsement and possibly more problematic functioning (lower scores) on objective measures.

### Response to Drug Recalls

In 2021, the United States accounted for over 46% of worldwide pharmaceutical sales and is the world’s leading consumer of pharmaceuticals (Health, Pharma and Medtech, 2022). A consequence of the extensive use of medicines is product recalls in the pharmaceutical industry, which have increased dramatically over the years (Dickinson, 2001). The U.S. Food and Drug Administration (FDA) can mandate industry-wide recalls when there is a perceived risk to human health (U.S. Food and Drug Administration, 2022), and such recalls are far more common than industry-generated recalls (Dickinson, 2001). As an example, in 2013 the FDA listed 59 different drugs on its website that were recalled, the majority of which were Class I recalls, meaning exposure to the drug or product is more likely to cause “serious adverse health consequences or death” (U.S. Food and Drug Administration, 2013). Previous research focused mainly on the demographics of those who respond to recalls, their attitudes toward the companies involved in the recall (e.g., Blasche et al., 2008), or the characteristics of those who fail to comply with recall notifications (e.g., Cohen et al., 2012). However, there is a dearth of research examining the psychological variables that influence responses to drug recalls. Thus, the current research will focus on individual differences in health anxiety, experimentally manipulated litigation potential, and their interaction with respect to respondents’ self-reported symptoms, cognitive performance, and physiological responses to a drug recall announcement.

### The Present Research

The present study represents an experimental design with one manipulated categorical predictor variable (litigation/no litigation) and one measured continuous predictor (health anxiety). Three measures of health anxiety were employed and the presence of a common external motivator (opportunity to join a class-action lawsuit) was the experimentally manipulated variable. The importance of controlling the financial incentive is that drug recalls provide a context in which malingering (i.e., feigning symptom endorsement for monetary gain) can occur, and this motivation is conceptually distinct from symptom endorsement due to the experience of health anxiety. Additionally, publicity about class action lawsuits implies that a drug or medical device is dangerous while simultaneously incentivizing adverse event reporting for the potential of monetary gain. We also subsequently collected data in a second sample regarding health anxiety and symptom endorsement without mentioning the FDA recall to determine the impact of the recall context itself.

The outcome variables of interest were self-reported symptom endorsement, objective cognitive performance, and objective physiological responding, and each of these were assessed within the context of a simulated FDA recall. Importantly, it is not known how financial incentives interact with health anxiety to impact adverse event symptom endorsement or cognitive and physiological outcomes.

To better understand these variables, relatively healthy individuals were recruited who would presumably have a low base rate of symptom experience and reporting. Moreover, the side effects for the recalled medications were contrived (i.e., there was no actual drug recall) and the medications all produce pain relief and have few side-effects, which should in fact counter symptom experience. These circumstances should make it easier to attribute any emergent effects to the variables under investigation. Of particular interest is whether the opportunity to litigate and health anxiety impact; (1) the endorsement of symptoms that are, due to the suggestive nature of the experimental procedure, related to the recalled drug (after controlling for reported usage and constructs related to health anxiety, such as depression and anxiety scores), (2) performance on a cognitive measure, and (3) physiological responding. Based on the extant literature, it is expected that health anxiety will have its most significant impact on subjective self-reported symptoms, and show a weaker relation to the objective measures of cognitive and physiological functioning.

It is well established that individual differences in health anxiety are linked to increases in self-reported symptoms, and that some situations can magnify health concern and symptom reporting. For example, Camerone et al. (2021) demonstrated that verbal suggestion could increase or decrease pain sensitivity (referred to as nocebo hyperalgesia and placebo analgesia, respectively) in young healthy participants, and greater anxiety levels correlated with enhanced nocebo response magnitude. However, the literature regarding the consequences for physiological and cognitive measures is more equivocal. As an illustration, consider how expectancy effects for adverse outcomes, conceptualized as nocebo responding, appear to result in strong effects when focusing on subjective, patient reported symptoms (e.g., Turi et al., 2018; Winkler and Hermann, 2019; Wolters et al., 2019), but those same effects are typically smaller for more objective outcomes such as third party-reported symptoms (Meissner, 2005). In closer alignment with the current research, Zech et al. (2020) showed that negative verbal suggestions (e.g., statements indicating an individual in a clinical setting will experience pain) lead to decreases on objective measures of physical strength; with anxiety seeming to enhance this effect. Similarly, researchers have shown that treatment expectations can impact motor performance, in the form of reduced force and increased fatigue, and that higher anxiety also plays an important role (Corsi et al., 2016).

Based on these findings and the previously discussed literature on financial incentives and their influence on symptom experience, the present study examined objective outcomes in addition to subjective self-perceptions of symptoms. We predict increases in physiological symptom experience (blood pressure and heart rate) and decreases in cognitive functioning (slower speed and more errors on an executive measure) as a product of health anxiety in the face of a drug recall, but with smaller effect sizes than will occur for the predicted increases in subjective symptom endorsement. It is also likely that a health anxious response set will converge with malingering in terms of heightened symptom endorsement, but the two may diverge with respect to the objective measures. Specifically, the literature suggests that health anxiety would result in greater effects (higher scores) on the physiological measures, whereas malingering may exert a greater influence (lower scores) on the cognitive measure; though both would result in poorer performance, with the effects of anxiety being unintentional and the effects of malingering being intentional. In fact, the expected effect size for the analyses involving monetary incentives and malingering should produce *r*-values ranging from 0.47 to 0.74 (see meta-analysis by Rohling et al., 1995; see also Meyer et al., 2001). The effect size estimate for a measure of health anxiety related to self-reported symptoms has been documented as *r* = 0.40 (Lecci et al., 1996). These are considered medium effect sizes (see Cohen, 1992). Smaller effects are generally expected for non-self-report outcomes such as physiological and cognitive measures (e.g., *r* = 0.20). To achieve a power of 0.80 for any main effects, the necessary sample size would range from 47 for the largest effect sizes to 194 for the smallest.

## Materials and Methods

### Participants

Participants were students at a university in the southeastern United States with an enrollment of approximately 15,000 at the time the data were collected. Participation satisfied research participation requirements for a General Psychology course and credit opportunities in other courses. The students represent majors from across the university. One hundred seventy-five participated, although 14 subjects were removed (10 were < 18 years, three had incomplete data, and one due to computer error). The remaining 161 participants (68% female) comprised the current sample. The mean age was 20 (*SD* = 5.56), 83% were Caucasian, 2.5% African American, 4% Hispanic, and 5.5% “other.” There were no additional exclusion criteria for this study.

### Measures

#### Self-Reported Health Anxiety

The *Minnesota Multiphasic Personality Inventory-2* (MMPI-2; Butcher et al., 2001) is a 567-item true/false questionnaire that is commonly used to measure personality and psychopathology in clinical settings with well-established validity and reliability. The MMPI-2 has scales assessing response tendencies (validity scales) and clinical scales (Graham, 2011). This study used the K-corrected scale 1 (Hypochondriasis; Hs) and scale 3 (Hysteria; Hy) scores. Scores on depression (D; scale 2) and anxiety (supplemental scale A) served as covariates. Although scales 1 and 3 are associated with increased levels of somatic symptom reporting, the endorsement of health-based symptomatology and those associated with psychopathological processes like health anxiety, are considered overlapping but distinct experiences and, as such, commonly utilized in medical assessments (Arbisi and Butcher, 2004). The MMPI-2 is one of the most widely used clinical measures in the field of psychology (Ball et al., 1994) and all scales are shown to have strong internal reliability (Hunsley et al., 1988). In the present study, the MMPI-2 scales 1 and 3 serve as a contrast to a measure that directly captures pure symptom worry, the Whitely Index.

#### The Whitely Index

The Whitely Index (Pilowsky, 1967) is a 14-item questionnaire rating the degree to which statements are true (1 = “Not at all” and five = “Extremely”). The Whitely Index assesses health fear/anxiety and includes questions such as “Are you bothered by many pains and aches?” and “Do you think that you worry about your health more than most people?” (Pilowsky, 1967, 1978). The Whitely Index’s test-retest reliability, convergent validity, and internal reliability have been established previously (Speckens et al., 1996).

### Cognitive Functioning

The Trail Making Test (TMT; BrainBaseline^®^ by Digital Artefacts^®^, Iowa City, IA, United States) was administered using a first-generation Apple^®^ iPad^®^. The TMT used in this study required participants to draw lines connecting numbers in numerical order from 1 to 2 to 3 (Part A). The software provided completion time, errors, and restarts (reinitiating a trial after a line tracing error). Performance on the trail-making test Part A has been interpreted by neuropsychologists as reflecting attention, visual search and scanning, psychomotor speed, and the ability to execute and modify a plan of action (Salthouse and Fristoe, 1995). (*Note*: The computer program also included Trails B. However, some participants were not given the proper directions for this task, especially when errors occurred, thereby creating unknown variability due to the instructional set. As a result, these scores are not presented.) The TMT is recognized as one of the most commonly used neuropsychological assessments (Rabin et al., 2016), it can be administered quickly on a computer for high levels of standardization and has also been used to detect malingering (e.g., Iverson et al., 2002).

### Physiological Functioning

The Omron Automatic Blood Pressure Monitor, Model HEM-7311, assessed blood pressure and pulse from the left arm while participants were seated with their left arm extended at a 90-degree angle with the body. Resting blood pressure and heart rate were recorded from a single digital readout by the research assistant. The Omron device is listed on the US Blood Pressure Validated Device Listing, and was calibrated at the start of the study with a second, automated blood pressure and heart rate device.

### Self-Reported Symptoms

Participants were asked whether they experienced any of the listed side effects/symptoms in response to the recalled medications, with ratings ranging from 0 (meaning “not at all”) to 3 (meaning “a great deal”). A sum of these ratings was computed. The symptoms included; difficulty sleeping, nausea, diarrhea or constipation, light-headedness, blurred vision, lower back pain, difficulty concentrating, difficulty breathing, rapid heart rate, and tingling in the extremities. Each of these symptoms is among the most reported by adults (Verbrugge and Ascione, 1987).

### Procedure

The study took place in the clinical research unit of a building operated by the university’s school of nursing within the college of health and human sciences, where students are trained to conduct clinical pharmaceutical research; thereby providing a realistic backdrop. Participants who volunteered were informed that they were taking part in a national FDA-funded study regarding a recent recall of over-the-counter pain medications. Signage indicating that this was an FDA field site was posted at the building entrance and in the hallway outside the lab. Eight female research assistants (RAs), who were advanced college undergraduate students, collected all of the data. The RAs had been members of the lab for at least one semester prior to the data collection, and received course credit for directed individual study. They were trained in the procedure over the course of 4 weeks and were monitored for consistency by a senior student who used the data for an honor’s thesis. Because the computer program randomly assigned participants to one of two litigation conditions that differed only with respect to the content of one part of the mock FDA webpage, the RAs would not have been privy to condition at the time the data were collected.

Participants first read and signed informed consent. The cover story for the study provided to participants was that the FDA was collecting information on the scope of the problem associated with the drug recall and the possible health consequences, especially among young, healthy adults. Participants were then directed to a professionally developed, mock version of the FDA website, which contained a link to information on a recent drug recall. The website was, in fact, hosted on a microcomputer that, unbeknownst to the participants, was not linked to the internet. The mock FDA website informed the participants that they had initiated a recall on commonly used pain medications due to aversive side effects. The recalled medications list included Tylenol, Extra Strength Tylenol, Tylenol PM, Tylenol Flu, Aleve, Aleve PM, Aleve Extended Release, Goody’s, Goody’s Extra Strength, BC, BC Arthritis, and Walmart brands of ibuprofen and acetaminophen. Possible side effects, ostensibly associated with taking these medications, were also listed (identical to the self-reported symptoms listed above).

Half of the participants were randomly assigned to a condition in which they were informed on the mock FDA website that there was a class-action lawsuit associated with the drug recall and that they could take part (litigation condition). The potential monetary compensation for the class action was stated to be between $46,300 and $1,000,000 depending on their symptoms, the size of the final award, and the number of people joining the class action. Individuals were asked to click a link to indicate their interest in entering the class action lawsuit. The remaining participants were assigned to a condition in which the website informed them that they could not litigate due to a Supreme Court decision (non-litigation condition).

Participants then indicated how often in the previous year they had used the named medications and rated how often they experienced specific aversive symptoms. Blood pressure and pulse were taken while the participant was seated at a table. Participants then completed the computerized TMT, which was described as a test of attention, and then completed the Whitely and the MMPI-2. Total testing time with each individual participant was between 90 and 120 min. Afterward, participants were fully debriefed and provided believability ratings (1 “completely” to 4 “not at all”). The procedure was approved by the host university’s institutional review board, and none of the data have been published elsewhere.

**FIGURE 1:**
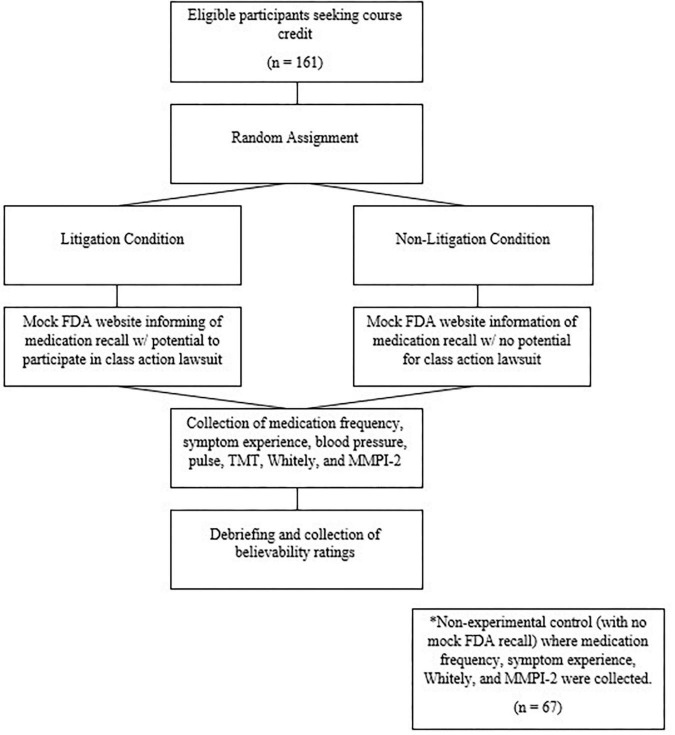
Flowchart describing the procedure.

Data were subsequently collected for a control condition. This *post hoc* data collection allowed us to examine the effects of the FDA context, though importantly, the participants were not randomized for this analysis. Sixty-seven participants completed the same self-report measures as noted above, but with no information regarding the FDA recall or litigation (i.e., FDA condition v. control, with these conditions coded +1 and –1, respectively). The 67 participants for this control (no FDA) condition had an average age of 19.41 years (*SD* = 3.21) and was 77.8% Caucasian. Age and gender did not differ significantly from those in the original sample. See Figure 1 for an overview.

All participants were awarded experimental credit for their psychology class in exchange for their time. The presented research was approved by the host institution’s Institutional Review Board (#H1011-160).

### Statistical Analyses

Hierarchical regression analyses were employed to examine the predictive value of health anxiety, the manipulated condition, and their interaction. The experimental condition was effect coded, with +1 denoting the litigation condition and –1 for the non-litigation condition. Similarly, when comparing the FDA context to the subsequently collected data with no FDA context, we employed effect coding (+1, –1, respectively). All other variables were centered within their respective distributions. An interaction term was created by multiplying the centered variables by the effect-coded condition, and follow-up probes were used to assess all interactions (Aiken and West, 1991). All analyses were conducted controlling for summed usage scores (the extent to which the medications were used), with this information entered in Step 1 of the regression. The main effects of health anxiety and the effect coded experimental condition were entered in Steps 2 and 3, respectively, and their interaction was entered in Step 4. Effect size estimates are reported in the form of r-square values and Cohen’s ds for the obtained results, and 95% confidence intervals are reported for the interactions. Small to medium effect sizes were estimated to emerge based on the literature, and G-Power was used to estimate the needed sample size to achieve statistical power of at least 0.80 (i.e., *N* > 150). Outlier analyses indicated no problematic values and there were no issues with multicollinearity. Correlations between the scales used to assess health anxiety are reported in Table 1.

**TABLE 1:** Correlations between individual difference measures of health anxiety.

	Hs	Hy	Whitely
Hs	___	0.75*	0.44*
Hy		___	0.38*
Whitely			___

*p < 0.01.

## Results

Most participants (80.4%) rated the FDA recall as “completely believable” (rated as 1) and 19.6% reported it as “believable” (rated as 2) with a mean rating of 1.2 (SD = 0.4) out of 4 (rated as “completely not believable”; 13 participants were not given this question). The data to follow report on all participants, as the findings are the same irrespective of the believability ratings, providing further support that participants held similar views with respect to the credibility of the experimental procedure.

### Predicting Self-Reported Side-Effect Symptom Experience

The mode and mean usage were “a few times per year” for 75% of the sample, and <15% of the sample reported weekly or daily usage. Symptom experience scores ranged from 0 to 47 (*M* = 14.7, *SD* = 9.1). Self-reported usage of the recalled medications accounted for 3.6% of the variance in symptom endorsement (Cohen’s *d* = 0.386) [*F*(1,159) = 5.90, *p* = 0.016], with higher use being associated with higher symptom endorsement.

After statistically controlling for usage, the Whitely Index (*b* = 0.47) accounted for the most variance in symptom endorsement at 21.7% (Cohen’s *d* = 1.053) [*F*_change_ (1,158) = 46.0, *p* < 0.001], the Hs scale (*b* = 0.41) accounted for 17.1% of variance (Cohen’s *d* = 0.908) [*F*_change_ (1,158) = 34.18, *p* < 0.001], and the Hy scale (*b* = 0.41) accounted for 16.5% (Cohen’s *d* = 0.889) [*F*_change_ (1,158) = 32.61, *p* < 0.001]. In all cases, higher scores resulted in greater symptom endorsement. The three measures of health anxiety were also simultaneously entered into the regression equation, yielding a total explained variance of 28.5% after controlling for usage (Cohen’s *d* = 1.263) [*F*_change_ (3,156) = 21.84, *p* < 0.001], with the Whitely accounting for the bulk of the variance in self-reported symptoms (*b* = 0.34, *t* = 4.62, *p* < 0.001).

Because depression and anxiety have been shown to significantly and substantially inflate retrospective accounts of physical symptoms (Howren and Suls, 2011), it is important to determine if the measures of health anxiety provide unique information. After statistically controlling for usage, the MMPI-2 scores of depression (clinical scale 2) and anxiety (supplemental scale A) together accounted for 12.1% of the variance in symptoms (Cohen’s *d* = 0.742) [*F*_change_ (2,157) = 11.23, *p* < 0.001], which is statistically significant and a substantial effect. Nevertheless, each of the three measures of health anxiety continue to significantly predict symptom endorsement over and above depression and anxiety, with the Whitely, Hs and Hy accounting for an additional 11.5% (Cohen’s *d* = 0.721) [*F*_change_ (1,156) = 24.63, *p* < 0.001], 12.6% (Cohen’s *d* = 0.759) [*F*_change_ (1,156) = 27.45, *p* < 0.001], and 12.7% [*F*_change_ (1,156) = 27.73, *p* < 0.001] of the variance, respectively. Moreover, when examined collectively, the three measures of health anxiety still account for an additional 19.7% (Cohen’s *d* = 0.991) [*F*_change_ (3,154) = 15.68, *p* < 0.001] of the variance in symptom reporting after controlling for usage and MMPI-2 depression and anxiety scores.

Condition (litigation vs. non-litigation) was not a significant predictor of symptom endorsement, and there were no significant interactions between health anxiety and condition (Table 2).

**TABLE 2:** Descriptive information for symptom endorsement by condition.

	Mean	*SD*
Non-Litigation	14.45	8.49
Litigation	15.08	11.72
Control	16.19	16.41

We also evaluated the effect of the FDA recall by comparing the full 167 participants to the subsequently collected 67 control participants. Medication usage did not differ significantly in this group as compared to the original sample. Using regression analyses it was also shown that there were no main effects for condition (exposure to mock FDA drug-recall vs. control) on self-reported symptoms (*F_change_* = 0.722, *p* = 0.396). Interactions between condition and each of the three measures of health anxiety also failed to reach significance for self-reported symptoms. Under these conditions, however, the total explained variance in self-reported symptoms for the three measures of health anxiety after controlling for usage was 12.6% (Cohen’s *d* = 0.759) [*F*_change_ (3,62) = 3.33, *p* = 0.025], which is less than half that obtained when there was an FDA recall context (which had 28.5% explained variance; Cohen’s *d* = 1.263). Thus, although still significant, the effect for the measures of health anxiety in this control condition is trending smaller; Fisher’s *z* = 1.52, *p* = 0.06.

### Predicting Cognitive Performance

Regression analyses were used to determine whether health anxiety and the litigation condition predict performance on the cognitive measure (restarts, errors, and completion time for TMT Trails A).

All measures of health anxiety significantly predicted restarts for TMT Trails A after controlling for usage. The Hy scale accounted for 6.9% of the variance in restarts (Cohen’s *d* = 0.544) [*F*_change_ (1,158) = 11.72, *p* = 0.001], the Hs scale accounted for 6.2% of the variance (Cohen’s *d* = 0.514) [*F*_change_ (1,158) = 10.44, *p* = 0.001], and the Whitely Index accounted for 4.1% of the variance (Cohen’s *d* = 0.414) [*F*_change_ (1,158) = 6.85, *p* = 0.01]. In all cases, higher scores on the measures of health anxiety resulted in more restarts (poorer performance).

**FIGURE 2:**
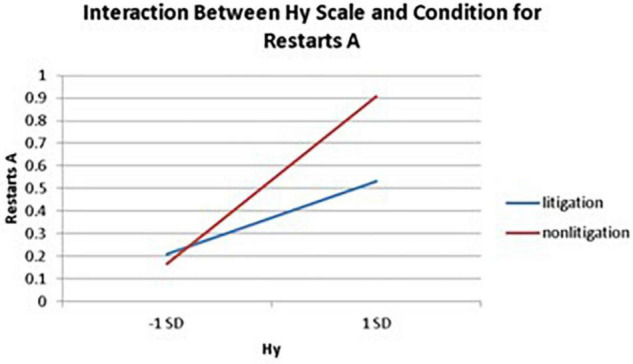
Plotting the interaction between the Hy scale and condition when predicting restarts for Trails A of the TMT.

**FIGURE 3:**
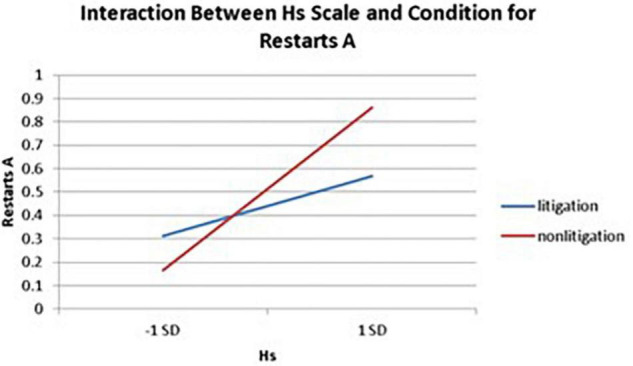
Predicting the interaction between the Hs scale and condition when predicting restarts on Trails A of the TMT.

**FIGURE 4:**
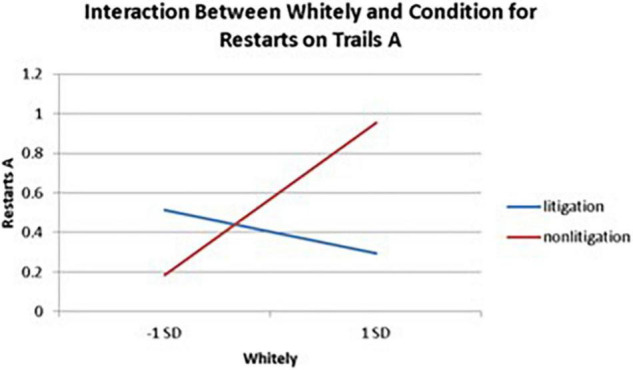
Plotting the interaction between Whitely and condition when predicting restarts on Trails A of the TMT.

Significant interactions between health anxiety and litigation condition also emerged when predicting restarts on Trails A (see Table 3 and Figures 2–4). To probe these interactions, values for participants who were low and high on the various measures of health anxiety were estimated. On each subscale, participants scoring 1 *SD* above the mean were identified as high in health anxiety and those scoring 1 *SD* below the mean were classified as low in health anxiety (Aiken and West, 1991). Simple slopes analyses revealed that as Hy {β = 0.37, *t*(86) = 3.64, *p* < 0.001, CI [–0.291, –0.015]}, Hs {β = 0.34, *t*(86) = 3.33, *p* < 0.001, CI [–0.279, –0.007]}, and Whitely {β = 0.39, *t*(86) = 3.77, *p* < 0.001, CI –0.354, –0.082]} scores increased, there were more restarts on Trails A for those in the non-litigation condition. In contrast, in the litigation condition, Hs [β = 0.13, *t*(73) = 1.10, *p* = 0.28], Hy [β = 0.16, *t* (73) = 1.39, *p* = 0.17], and Whitely [β = –0.02, *t*(73) = –0.16, *p* = 0.87] scores were not related to restarts.

**TABLE 3:** Significant interactions for cognitive performance on Trails A outcomes.

Outcome measure	Predictor	*R* ^2^_change_	*F*_change_	β	*SE*(β)	95% confidence interval	*p-value*
Restarts	Hy × Con	0.027	4.82	–0.153	0.070	–0.291, –0.015	0.030
Restarts	Hs × Con	0.024	4.25	–0.143	0.069	–0.279, –0.007	0.041
Restarts	Whit × Con	0.057	10.0	–0.218	0.069	–0.354, –0.082	0.002
Errors	Hs × Con	0.039	6.37	–1.183	0.469	–2.109, –0.257	0.013
Errors	Whit × Con	0.033	5.42	–1.078	0.463	–1.993, –0.164	0.021
Completion Time	Hs × Con	0.018	3.07	–2106.0	1201.63	–4478.90, 266.96	0.082

*R-square change values for Hy, Hs, and Whitely increase on restarts in all cases to 0.031, 0.03, and 0.059, respectively, after statistically controlling for depression and anxiety scores.*

**FIGURE 5:**
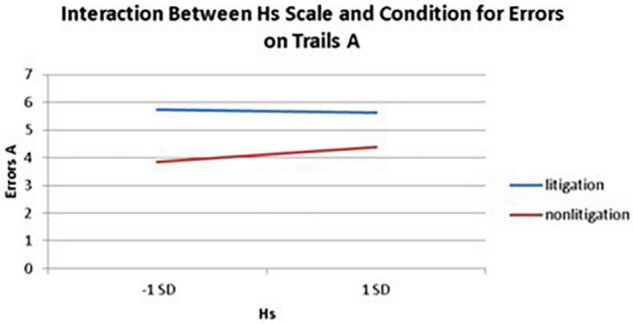
Predicting the interaction between the Hs scale and condition when predicting errors on Trails A of the TMT.

**FIGURE 6:**
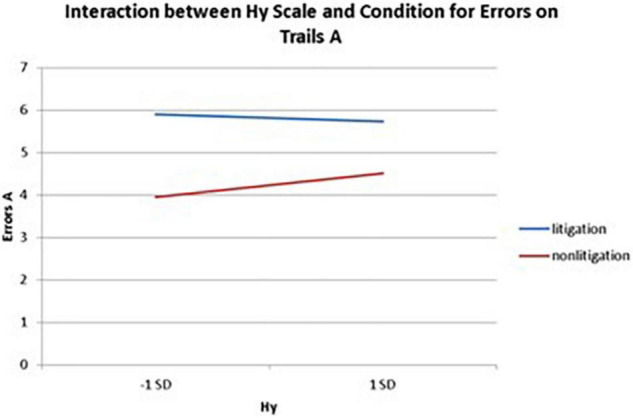
Plotting the interaction between Hy scale and condition when predicting errors on Trails A of the TMT.

Interactions also emerged between litigation condition and two of three health anxiety measures when predicting errors for Trails A, after controlling for usage (see Table 3 and Figures 5, 6). To probe these interactions, values for participants who were low and high on the health anxiety were estimated at 1 *SD* above and below the mean of each subscale, respectively. Simple slopes analyses revealed that as Hs {β = 0.27, *t*(86) = 2.52, *p* = 0.013, CI [–1.993, –0.164]} and Hy (β = 0.28, *t* = 2.67, *p* = 0.009, CI [–2.109, –0.257]) scores increased, there was an increase in Trails A errors for the non-litigation condition. Whereas for the litigation condition, Hs [β = –0.06, *t(73)* = –0.49, *p* = 0.62] and Hy [β = –0.08, *t* = –0.66, *p* = 0.52] scores were unrelated to errors.

All measures of health anxiety significantly predicted the time of completion for Trails A. The Hy scale accounted for 7.7% of the variability in completion time (Cohen’s *d* = 0.578) [*F*_change_ (1,158) = 13.26, *p* < 0.001], the Hs scale predicted 6% (Cohen’s *d* = 0.505) [*F*_change_ (1,158) = 10.18, *p* = 0.002], and the Whitely Index accounted for 3.9% of the variability in time (Cohen’s *d* = 0.403) [*F*_change_ (1,158) = 6.39, *p* = 0.012]. In all cases, as health anxiety increased, participants took longer to complete Trails A. No significant effects for time of completion emerged for the litigation condition. The interaction for the Whitely scores and condition was also not significant, but the interaction between condition and Hs approached significance. The latter was characterized by the same pattern of simple slopes as seen in the previous analyses (i.e., significant positive beta weight, but only for the non-litigation condition when predicting time to complete Trails A; see Table 3).

Thus, health anxiety was consistently associated with poorer performance on the TMT (slower time, and more errors and restarts), but only in the non-litigation condition.

### Predicting Physiological Functioning

Table 4 provides descriptive information for the physiological measures. The Hs [*F*_change_ (1,155) = 5.85, *p* = 0.017] and Hy [*F*_change_ (1,155) = 5.45, *p* = 0.021] scales accounted for 3.6% (Cohen’s *d* = 0.386) and 3.4% (Cohen’s *d* = 0.375), respectively, of the variance in systolic blood pressure after controlling for usage. Beta weights were negative, indicating that as scores on the scales increased, systolic blood pressure decreased. Only the Hy scale predicted diastolic blood pressure, accounting for 3.4% of the variability (Cohen’s *d* = 0.375) [*F*_change_ (1,155) = 5.52, *p* = 0.02] with a negative beta weight. The Whitely scale did not predict any of the physiological measures.

**TABLE 4:** Descriptive information for the physiological measures.

	Mean	*SD*	Range
Systolic BP	107.6	12.5	77140
Diastolic BP	67.8	8.8	51–107
Heart rate (pulse)	73.5	12.7	47–119

*Systolic and diastolic BPs correlated 0.29, p < 0.001. BP measures and heart rate were not significantly correlated with each other.*

The Hs scale significantly predicted 4.8% of the variance in heart rate after accounting for usage (Cohen’s *d* = 0.449) [*F*_change_ (1,155) = 7.73, *p* = 0.006]. The positive beta weight indicates that as scores on Hs increased, so did the participants’ heart rate.

There were no effects for the litigation condition on the physiological outcomes, and no interactions were found between the measures of health anxiety and condition for the physiological measures.

## Discussion

The present research indicates that health anxiety consistently predicts symptom endorsement, and this association is independent of depression and general anxiety. It was also shown that all measures of health anxiety predicted self-reported symptom experience, but symptom endorsement was unaffected by the presence of financial incentives, and only minimally affected by self-reported usage (the latter is to be expected given the fabricated nature of the drug recall). Although MMPI-2 measures of depression and anxiety were also related to symptom endorsement, these psychological variables were markedly less predictive, suggesting health anxiety has a unique association to symptom reporting beyond more general negative emotional states (see also Lecci et al., 1996). Finally, although the measures of health anxiety predicted symptom endorsement outside the context of the FDA recall (as found in the subsequent data collected in a non-randomized control condition), their predictive ability trended downward; as the *R*-square value was less than half in size. This raises the possibility that health anxiety-related symptom endorsement may be modestly amplified within the context of a drug recall announcement, though this would need to be examined in a purposely designed experiment. Notably, the obtained effects for health anxiety resulted in moderate to large effect sizes (Cohen, 1992), with each measure accounting for 16.5–21.7% of the variance, and when combined, predicting more than 28% of the variance in symptom endorsements (equating to a large Cohen’s *d* = 1.25). Because the FDA recall and the adverse effects from the medications were wholly fabricated, and because similar findings emerged in the non-FDA condition, we can conclude that the driving mechanism behind the reported symptoms could not be the medications themselves, especially given that the use of medications was low and statistically controlled. Thus, symptom endorsement must be associated with genuine symptom *perception* or malingering (i.e., intentional over-reporting for the sake of compensation). The high proportion of variance in symptom endorsement accounted for by health anxiety, and the limited effect of the experimentally controlled financial incentive suggests genuine symptom perception driven by health anxiety is the most prominent mechanism, beyond any tendency for general over-reporting. Importantly, usage should not be a strong predictor because the side effects were not actually related to the medications, and usage rates were low. This is an essential aspect of this experimental paradigm, as real-life drug recalls would necessarily confound usage (i.e., those responding to a recall notice would be those using the drugs), *a priori* symptoms, which may be higher in those who take medications, and actual medication side effects.

Consistent with the literature, using health anxiety to predict subjective outcomes (self-reported symptoms) resulted in larger effects relative to the prediction of objective cognitive and physiological measures (e.g., Drici et al., 1995; Beedie et al., 2008). However, the current findings add to the literature by illustrating; (1) an effect even for young, healthy individuals, (2) the extensive impact of health anxiety (large effect sizes) when the context is methodologically controlled, and (3) interactions between health anxiety and external incentives (litigation) when examining a cognitive outcome. Thus, when considering responses to an FDA recall, the resulting effects appear to depend upon how the sequelae are quantified (i.e., which outcome variable is considered).

With respect to the interactions between health anxiety and litigation potential, it was found that greater health anxiety typically resulted in more problematic TMT (cognitive) performance in the non-litigation condition, whereas health anxiety was unrelated to TMT performance in the litigation condition. This finding may have some implications for differentiating a health anxiety-driven response from a malingering response when, for example, dealing with individuals who are falsely claiming to experience cognitive difficulties in the context of a lawsuit (see section “Implications”).

In the present study, MMPI-2 measures of health anxiety (Hs and Hy) were associated with lower scores on at least one of the blood pressure readings, and Hs scores were associated with increased heart rate. One possible explanation for the latter finding is that an acute stress response is prompted by the fear of what the aversive symptoms could mean. Activation of the sympathetic autonomic nervous system, part of the “flight or fight” response, is well documented and is known to cause an increase in heart rate, as well as other changes (Jansen et al., 1995). Of course, what is unknown is whether the observed physiological differences are durable or simply reflect a short-term response. It is more difficult to explain why those participants with higher health anxiety displayed a decrease in their systolic blood pressure, as previous research has not shown this effect. However, the current results do suggest that there may be tangible physiological changes associated with the measures of health anxiety, indicating effects that extend beyond self-reported symptom endorsement to changes that may be less apparent to the individuals experiencing them.

### Implications

When notifying the public about FDA recalls, it is general practice to list adverse responses (symptoms) that are thought to be associated with the recalled medication. From an ideal standpoint, the recall should activate risk perceptions for those who have used the medications, as well as activating mental representations of coping procedures that are linked to specific actions, such as discontinuing use of the medications and following up with any resulting symptoms (see the common-sense model of self-regulating health and illness; Leventhal et al., 2003). However, those with high health anxiety may be especially attuned to information about adverse effects and more prone to experience changes in their health perceptions (Salkovskis and Warwick, 2001; Lecci and Cohen, 2002). Although announcing the symptoms associated with a drug recall may inadvertently negatively impact the perceived health (i.e., trigger symptom reporting) of individuals with high health anxiety independent of whether participants even took the medication, it also appears to be the case that health anxiety predicts symptom reporting without the pretext of an FDA recall. The latter finding is in keeping with the literature that links health anxiety with a wide range of chronic medical conditions and symptom experiences (see review by Lebel et al., 2020). Similar findings may also occur for broader related constructs such as negative affectivity (Watson and Pennebaker, 1989), as this construct has also been linked to broad symptom endorsement (e.g., Barsky, 1992; Karoly and Lecci, 1993). Indeed, the revised symptom perception hypothesis explicitly predicts that the negative emotional states of anxiety and depression uniquely and powerfully influence retrospective reports of physical symptoms (Howren and Suls, 2011), and this would be in keeping with how symptoms were reported in the current study. However, in the current research, measures of health anxiety remained significant and substantial predictors even after statistically controlling for depression and anxiety scores. Thus, although constructs reflecting broad negative emotional states undoubtedly play a role in predicting symptom endorsement, there remains a substantial effect for the experience of health anxiety in symptom reporting (see also Barsky et al., 2002).

Another potential implication of this research is in the arena of pharmaceutical development. The Code of Federal Regulations (45CFR46) mandates the safety of individuals who participate in research, and consistent with this mandate, all four phases of FDA approved clinical trials involve close monitoring of the side effects and adverse events (e.g., National Institutes of Health, 1999). The U.S. Food and Drug Administration (2010) defines adverse events quite broadly as “any untoward medical occurrence associated with the use of a drug, whether or not considered drug-related” (p. 7). Thus, even the perception of symptom experiences may be sufficient to be considered an adverse event. Consequently, the presence of health anxious individuals in a drug trial could increase the likelihood that otherwise safe medications are identified as having too many adverse events. Health anxiety may also be responsible for the common occurrence of a subset of symptoms that appear as side effects for many medications. Health anxiety may also create noise in the identification of genuine drug effects. Because medication effect sizes are typically classified as small to extremely small (Cohen, 1992; Cuijpers et al., 2010; Bartolucci et al., 2011; Sullivan and Feinn, 2012; Mustian et al., 2017), excessive adverse symptom reporting could introduce “noise” to the measurement of genuine drug effects that can affect the precision of measuring pharmaceutical effects. Thus, it may be helpful to consider either screening out those with elevated health anxiety from investigative trials or weighing their information less (e.g., using scores on measures of health anxiety as a covariate) so as not to unduly influence the determination of adverse effects associated with the drug under investigation.

It is also essential to note that the endorsement of symptoms in healthy participants required very little symptom information when it was given in an official context. This raises the question of how these results impact actual drug recalls. Interestingly, when offered the opportunity to partake in a class-action lawsuit, all but one participant agreed to do so despite the fabricated symptoms associated with the FDA recall. This indicates a high willingness to join class-action lawsuits even in cases where there is no possibility that those joining the suit have experienced adverse effects caused by the product. Although this has implications for malingering, it must be acknowledged that there was no main effect for the potential to litigate for any of the measures. Moreover, high health anxiety resulted in a performance that was atypical for malingering on the cognitive measure, as more problematic scores emerged in the non-litigation condition as compared to the litigation condition. Thus, the observed findings are more complex in nature, and a health anxiety response-set may be distinct from a malingering response-set. [*Note:* this conclusion is further bolstered by the fact that MMPI-2 measures that have been used to assess possible malingering, *F* scale and scale 4 (Pd) scores, account for markedly less variance in symptom endorsement after usage; only 5.9% combined, and do not account for significant variance in Trails A scores. Moreover, the three measures of health anxiety explain an *additional* 22.8% of the variance after entering the *F* scale and scale 4 scores; *F*_change 3,154_ = 17.34, *p* < 0.001.]

Finally, the current findings may have implications that go beyond the influence of health anxiety on symptoms endorsement. For example, nocebo responses can occur when expectations of adverse outcomes of medical treatments or agents produce negative or worsening health symptoms (Benedetti et al., 2007; Mitsikostas et al., 2020). Beliefs about risks, expectations of specific symptoms and anxiety are known to increase nocebo responses (Colloca and Benedetti, 2007; Cocco, 2009; Colloca and Miller, 2011; Daniali and Flaten, 2021), and information about adverse effects can trigger nocebo responses (Bagarić et al., 2021). For example, negative messaging by health professionals about side-effects increases reports of cognitive problems in people receiving chemotherapy (Jacobs et al., 2017), and media reports about WiFi radiation can increase reports of somatic symptoms (Bräscher et al., 2017). Similarly, it has been established that adverse event reporting related to vaccination is associated with news coverage and internet database search numbers in the concurrent month (Faasse et al., 2017). In their review of the mechanisms underlying nocebo responding, Benedetti et al. (2007) suggested that anticipatory anxiety aroused by information plays a significant causal role. Thus, the response to highly publicized drug recalls and lawsuits could be conceptualized as a form of nocebo responding (involving anticipatory health anxiety), and future research could explore this theoretical connection more deeply.

Historically, differentiating between health-anxious somatic responses and malingered responses has proven difficult, despite notable differences in causality (i.e., somatic responses being related to genuine symptom perception and malingering being associated with feigned symptom experience; Bellamy, 1997). “Compensation neurosis” was once offered as an intermediate explanation for these phenomena when individuals experience exaggerated symptoms when faced with the stress of seeking financial compensation, but has lost favor due to the emergence of newer health-related DSM disorders (Hall and Hall, 2012). The current research offers deeper insight into how these phenomena differ during an FDA recall, with individual differences in health anxiety having much more pronounced effects than the potential for financial compensation.

### Limitations and Future Directions

The studied population may have impacted the results. College students are young and healthy and tend to use pain medication sparingly relative to older individuals and clinical populations. Indeed, the usage data from the current study indicate that 75% of participants only used pain medications a few times per year and in relatively small doses. A future study could focus on the elderly or those with chronic pain, where the usage of pain medication would be much higher. Because we did not collect information on the incidence of other medical conditions, general health, or medications to treat any conditions, this may confound our findings, and future research could collect such data. Future studies could also focus on the college population but target medications that are more commonly used by that group, such as attention deficit disorder, asthma, and depression medications. It is expected that by focusing on populations or medications with higher rates of use, the emergent effects could be more prominent, because the attribution of symptoms to those medications would plausibly be more extensive. In the current study, we addressed this issue by statistically controlling for medication usage. However, it is essential to recognize that studying a young and healthy population can be advantageous in that it reduces confounds that complicate the analysis with older and/or less healthy individuals. For example, those who actually take pain medications are likely experiencing more pain and other related symptoms like depression, and could be experiencing more side effects from medications. They also may be more likely to believe they are entitled to some compensation for their symptoms. Thus, although a college student sample necessarily undermines the generalizability of the findings, it provides added control over potentially confounding variables, as this group would have fewer confounding health conditions and would be less likely to take pain medications on a regular basis (e.g., Wensing et al., 2001; Green et al., 2016).

The current study did not control for the cognitive abilities of the participants, and this could have impacted scores on the cognitive measure. Of course, this would be more problematic for our measured variables (e.g., health anxiety), but less so for the experimentally manipulated variable (e.g., litigation potential), as the latter involved random assignment and presumably an unbiased allocation of cognitive abilities.

Ecological validity is always a concern in research. Although we likely mimicked an FDA recall, the cognitive and physiological assessments were less ecologically valid, and the experimental nature of this research is distinct from naturally occurring symptoms outside the lab. Health anxiety does predict symptom endorsement in general, though effect sizes tend to be smaller (e.g., Feldman et al., 1999). However, the experimental nature of this research and the use of a simulated drug recall are instrumental in isolating the impact of litigation potential on health anxiety. Notably, a manipulation check indicated participants believed they were participating in a genuine FDA drug recall, suggesting a relatively high level of ecological validity in the current research, despite its experimental nature. Similarly, it could be argued that retrospective symptom reporting is subject to biased recall. Class-action lawsuits in response to drug recalls, however, rely on similar types of reporting (i.e., the participation of individuals who have taken medications over some past period of time), meaning the current study’s retrospective nature is ecologically valid, at least with respect to the drug recall context.

Similar research has demonstrated an association between chronic pain and personality types characterized as “hypochondriacal” (Johansson and Lindberg, 2000) or “neurotic” (Ramirez-Maestre et al., 2004). The current research could be seen as building on these findings by suggesting that this association may be due, at least in part, to the propensity to *perceive* pain rather than being fully explained by the nociceptive input. Much like the previously discussed concept of “compensation neurosis,” the currently reviewed literature reflects a modernization in terminology and conceptualization (from “hypochondriasis” to “health anxiety”), which parallels the evolving nomenclature from the DSM-IV to the DSM-5.

An additional sample with no FDA information was collected after the completion of the original study in an attempt to provide some degree of control for the FDA context. Obviously, this is less than ideal, as there would be other potential systematic differences, and assignment to the FDA/no FDA context was not randomized. This necessarily limits our conclusions regarding the FDA context itself, as confounding variables such as participant maturation effects, could attenuate our ability to detect differences between these conditions.

Although we did obtain some findings of significance with respect to the physiological measures, it is noted that the measurement of blood pressure and heart rate can be less accurate when it is only measured once (e.g., Whelton et al., 2017). Similarly, additional cognitive data could be collected to provide convergent validity and improve measurement accuracy. Even the inclusion of TMT B data as a standalone or in combination with TMT A data could be used to generate index scores (e.g., difference scores and/or ratios) that could provide additional sensitivity to the consequences for cognitive functioning (see Tyburski et al., 2020). Unfortunately, the TMT B data were unavailable for the current study.

Finally, there is significant overlap between the common symptoms presented in the current research and symptoms associated with anxiety. For example, of the nine common side effect symptoms considered in the current study, six are recognized as generalized anxiety symptoms (Steer and Beck, 1997); and similar claims could be made of the study’s physiological measures (blood pressure and heart rate). This overlap in symptoms may further confound the differentiation of health anxiety-based effects from real medication side effects in health-anxious individuals. Importantly though, the current research shows that measures of cognitive functioning, like the TMT, were impacted by health anxiety. Because TMT performance is not typically associated with anxiety (e.g., Waldstein et al., 1997), it is reasonable to assume the studied drug recall effects are unique from those solely associated with anxiety responses. Future research, however, may wish to employ additional measures to parse out the influence of anxiety responses.

## Conclusion

This experimental study sheds light on the relationship between health anxiety and symptom endorsement, cognitive performance, and physiological functioning in the context of an FDA drug recall announcement; and few studies to date have explored the psychological variables at play under such circumstances. Of particular note, symptom endorsement was strongly predicted by health anxiety and these effects remain strong after statistically controlling for depression and anxiety. Even objective outcomes such as blood pressure, heart rate, and cognitive performance were modestly predicted by health anxiety, but not by the litigation condition. And interactions consistently emerged for the cognitive task, with generally poorer performance for those with higher health anxiety in the non-litigation condition; whereas health anxiety was unrelated to performance for the litigation condition.

In short, the present research and the general literature suggest that there are likely to be numerous, complex, and interacting factors that influence how individuals react to health-related information in the context of a drug recall. Importantly, individual differences in health anxiety appear to merit further attention not simply for self-reported data but also for what are considered more objective outcomes. This is in keeping with a trend in the literature indicating that how health-related phenomena are *perceived* is at least as important as the phenomenon itself, even with respect to physiological responses (see Crum and Langer, 2007; Benedetti et al., 2011). Health anxiety-based effects have the potential to decrease precision in the context of drug recalls, making it increasingly difficult to distinguish those whose symptoms are the result of genuine drug effects, those whose symptoms are related to the drug recall, and those whose responses are motivated by the potential to litigate. Although it is general practice in drug recalls to list potential adverse side effects caused by the medications in question, this may elicit unintended symptom experiences and health anxious individuals may be more susceptible. Thus, further consideration of health anxiety, perceived health, and their interactions with situational factors is indicated in better understanding how individuals respond to drug recalls.

## Data Availability Statement

The datasets presented in this study can be found in online repositories. The names of the repository/repositories and accession number(s) can be found below: https://osf.io/yhdz6/.

## Ethics Statement

The studies involving human participants were reviewed and approved by University of North Carolina Wilmington Institutional Review Board. The patients/participants provided their written informed consent to participate in this study.

## Author Contributions

SN conducted the study under the supervision of LL. AR was involved in collecting data for the control condition and completed related analysis. JK was involved in generating the idea of the study, served on the thesis committee, and assisted manuscript preparation. GP conducted nocebo-related literature review and conceptualization, contributed significantly to manuscript preparation, edited and conducted analyses, and prepared the manuscript for submission. LL assisted with editing throughout. All authors contributed to the article and approved the submitted version.

## Conflict of Interest

The authors declare that the research was conducted in the absence of any commercial or financial relationships that could be construed as a potential conflict of interest.

## Publisher’s Note

All claims expressed in this article are solely those of the authors and do not necessarily represent those of their affiliated organizations, or those of the publisher, the editors and the reviewers. Any product that may be evaluated in this article, or claim that may be made by its manufacturer, is not guaranteed or endorsed by the publisher.
